# The adverse impact of cytomegalovirus infection on intensive care units outcomes in critically ill COVID-19 patients: a single-center prospective observational study

**DOI:** 10.1007/s15010-025-02499-8

**Published:** 2025-03-19

**Authors:** Marina López-Olivencia, Raúl de Pablo, Noemí Paredes de Dios, Susana García-Plaza, Sergio Sáez-Noguero, Javier Sáez de la Fuente, Jesús Fortún, María Cruz Soriano Cuesta

**Affiliations:** 1 Department of Intensive Care Medicine, Ramón y Cajal University Hospital, Madrid, Spain; 2 Department of Pharmacy, Ramón y Cajal University Hospital, Madrid, Spain; 3 Department of Infectious Diseases, Ramón y Cajal University Hospital, Madrid, Spain; 4 Department of Medicine and Medical Specialties, University of Alcalá, Madrid, Spain

**Keywords:** Cytomegalovirus infections, COVID-19, Pneumonia viral, Active surveillance, Critical illness, Intensive care units

## Abstract

**Purpose:**

To assess the incidence and clinical impact of CMV infection in critically ill COVID-19 patients, examining ICU and hospital mortality, and length of hospital stay.

**Methods:**

In this single-center, prospective observational study (March 2020 - September 2022), 431 patients with COVID-19 pneumonia and moderate to severe ARDS were included. An active CMV surveillance protocol was implemented, analyzing CMV DNA in plasma and bronchoalveolar lavage (BAL). Clinical characteristics and outcomes were compared between CMV-COVID co-infected patients and those without CMV reactivation.

**Results:**

CMV-COVID co-infection was detected in 14.8% (64/431) of the cohort. Patients with CMV-COVID co-infection exhibited significantly higher ICU mortality (43.8% vs. 13.6%; *p* < 0.001) and hospital mortality (48.4% vs. 13.6%; *p* < 0.001) compared to patients without CMV. CMV infection was an independent predictor of hospital mortality (OR 4.91; 95% CI 2.76–8.75; *p* = 0.019). Earlier CMV reactivation was associated with an increased risk of hospital mortality (HR = 0.94; 95% CI: 0.90–0.98; *p* = 0.003). Additionally, CMV-COVID patients had a higher incidence of ICU-acquired infections and a prolonged hospital stay.

**Conclusions:**

In critically ill patients with SARS-CoV-2 pneumonia, CMV infection was frequently observed, and associated with increased ICU and hospital mortality. CMV co-infection correlated with a higher incidence of ICU-acquired bacterial and fungal infections and prolonged hospital stays. This emphasizes the importance of early CMV monitoring upon ICU admission, as timely detection and intervention could potentially mitigate its impact on patient outcomes.

## Introduction

Cytomegalovirus (CMV) reactivation occurs in one-third of CMV-seropositive patients during their stay in the intensive care unit (ICU) [1]. Patients with prolonged ICU stay, severe sepsis, and critical illness are at higher risk of developing CMV infection [2]. This risk may be increased in patients with severe pneumonia caused by SARS-CoV-2, due to infection-induced immunoparalysis and the use of immunosuppressive treatments and corticosteroids [3]. There are no definitions for the clinical entities, specific protocols, or data on the impact of CMV infection in this population [4]. This highlights the need to establish a consensus on the management of this virus and to develop protocols for active surveillance.

The main goal of this study is to analyze the incidence and clinical impact of CMV infection in its various clinical forms, in patients with SARS-CoV-2 infection admitted to the ICU.

## Materials and methods

### Study design and population

This prospective observational study was conducted from March 2020 to September 2022 in a 24-bed COVID-ICU at the Ramón y Cajal University Hospital in Madrid (Spain). We included all patients admitted to the ICU with a confirmed diagnosis of SARS-CoV-2 pneumonia by polymerase chain reaction or equivalent, and with moderate or severe acute respiratory distress syndrome (ARDS) criteria, according to the Berlin definition [5]. Patients who died within the first 48 h, who did not provide informed consent and those under the age of 18 were excluded. In this study, an active CMV infection surveillance protocol was implemented in the ICU, which consisted of twice-weekly quantitative analysis of CMV DNA in plasma and if there were clinical signs compatible with respiratory coinfection in bronchoalveolar lavage (BAL) too [6].

#### Definitions of clinical entities of CMV infection in patients with SARS-CoV-2 pneumonia (CMV-COVID group)

CMV infection encompasses three clinical entities: CMV viremia, probable CMV syndrome, and CMV disease, which are defined as follows:

##### COVID + CMV viremia

Detection of CMV DNA in plasma samples [7] using nucleic acid amplification, calibrated with the international standard (IU/ml) [8]. Plasma Limit of Detection (LOD) is 30 IU/ml, using the ABBOT ALINITY m™ CMV Assay, in patients with SARS-CoV-2 pneumonia.

##### COVID + Probable CMV syndrome

Fever and elevated C-reactive protein not explained by another cause, along with the detection of CMV in plasma, in patients with SARS-CoV-2 pneumonia. This definition is adapted from that used for solid organ transplant (SOT) recipients [9].

##### COVID + CMV disease

Involvement of a target organ (lung, intestine, liver, etc.) in patients with SARS-CoV-2 pneumonia, categorized according to the CMV Drug Development Forum definitions [7]:

##### Proven CMV pneumonia

Symptoms and/or signs of pneumonia along with the detection of CMV in lung tissue.

##### Probable CMV pneumonia

Signs and symptoms of pneumonia along with detection of CMV DNA in BAL.

##### Possible CMV pneumonia

High clinical suspicion of pneumonia, with CMV replication in blood.

The clinical management of SARS-CoV-2 infection was carried out following international guidelines [10, 11], including the use of corticosteroids and immunomodulators as part of the treatment [12]. Targeted treatment with ganciclovir or foscarnet was initiated for patients with probable CMV syndrome and CMV disease.

ICU-acquired superinfections, defined according to the main international guidelines and classified based on the type of microorganism [13, 14]. ECMM/ISHAM consensus criteria were used for CAPA (COVID-19 Associated Pulmonary Aspergillosis) case definitions [15].

### Data collection and impact analysis in CMV-COVID patients

Demographic data, immunosuppression status, severity scores at ICU admission (Sequential Organ Failure Assessment [SOFA] score, Simplified Acute Physiology Score II [SAPS II], and Acute Physiology and Chronic Health Evaluation II [APACHE II]), SOFA score on the day of CMV reactivation, the need for mechanical ventilation, Immunosuppressed treatment received and ICU-acquired superinfections were collected. To assess the impact of CMV infection in patients with SARS-CoV-2 infection (CMV-COVID), ICU and hospital length of stay, as well as ICU and hospital mortality, were analyzed in both, CMV-COVID and no CMV PATIENT. A multivariable analysis of overall mortality risk was performed, adjusting for risk factors such as age, average length of stay, and severity scores.

CMV viral load was assessed in both plasma and BAL.

Clinical data were retrieved from the institutional clinical database and managed using the REDCap^®^ (Research Electronic Data Capture) tool, available at IRYCIS (Ramón y Cajal Institute for Health Research).

The study protocol was approved by the institutional Clinical Research Ethics Committee of Ramón y Cajal University Hospital in March 2020 (Record No. 384).

### Statistical analysis

Statistical analysis was performed using SPSS V25.0^®^ (IBM^®^ Armonk, New York, USA) for Windows^®^. Data were expressed as mean ± standard deviation (S.D) or percentages as appropriate. Since most variables did not always fulfill the normality hypothesis, we compared continuous data by the Mann-Whitney U test and categorical data by Chi-square or Fisher’s exact test as appropriate. We also assessed risk factors for hospital mortality by multivariable logistic regression analysis. A stepwise backward-selection logistic regression analysis was performed to study the association with outcome. Variable selection was done based on p values < 0.10 and hospital mortality was the dependent variable. The level of statistical significance was set at *p* < 0.05.

Additionally, a time-dependent Cox regression analysis was conducted to evaluate risk factors for hospital mortality, including CMV reactivation as a time-dependent covariate. In cases of collinearity, affected variables were excluded from the final model. Results were reported as odds ratios (OR) or hazard ratios (HR) with 95% confidence intervals, considering *p* < 0.05 as statistically significant.

Multiple imputation was used to address missing data.

## Results

**Fig. 1:**
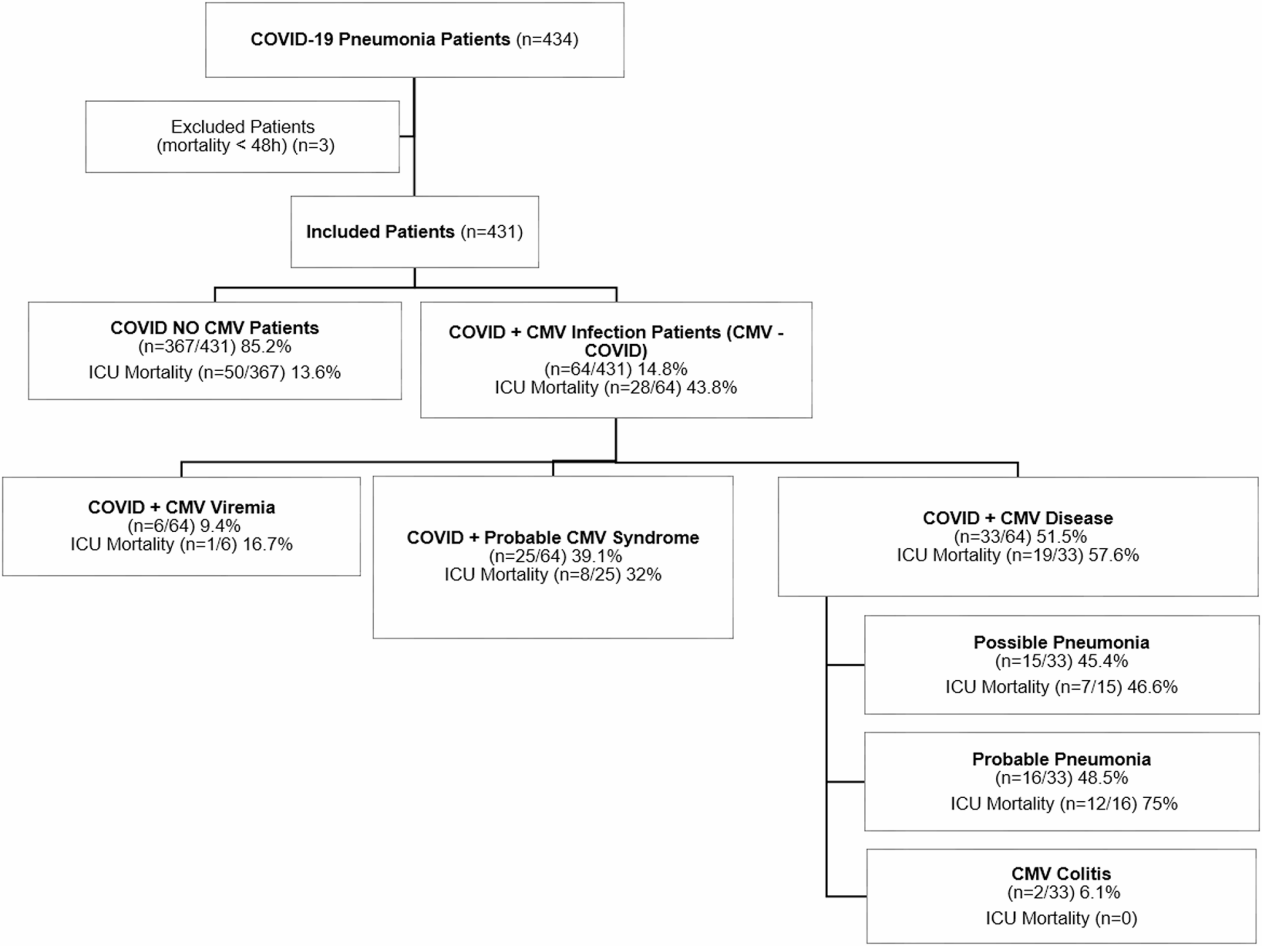
Flow diagram of patients’ disposition. COVID + CMV viremia is defined as detection of CMV DNA in plasma. COVID + Probable CMV Syndrome includes fever and elevated CRP with CMVdetected in plasma. COVID CMV Disease involves target organ infection (e.g., lung), classified as Probable (CMV DNA in BAL) or Possible (High clinical suspicion of pneumonia with CMVreplication in blood). Data are shown as number (percentage). Abbreviations: CMV: Cytomegalovirus; COVID: Coronavirus Disease; ICU: Intensive Care Unit

Between March 2020 and September 2022, 431 patients diagnosed with COVID-19 pneumonia met the inclusion criteria. The incidence of CMV-COVID co-infection was 14.8% (64 of 431 patients) (Fig. 1).

- In the comparison of characteristics between the two study groups, revealing significant differences in clinical outcomes (Table 1).

**Table 1:** Characteristics of patients by study group (Baseline Characteristics)

Variable	ALL *n* = 431	CMV-COVID GROUP *n* = 64	NO CMV COVID GROUP *n* = 367	*p* value
Gender (male)	298 (69.1%)	50 (78.1%)	248 (67.5%)	< 0.001
Age†	59.5 ± 12.0	65.6 ± 8.3	58.1 ± 12.9	< 0.001
APACHE II score†	15.8 ± 7.9	18.1 ± 7.3	15.3 ± 8.0	0.004
SAPS II score†	37.7 ± 15.7	42.3 ± 14.4	36.6 ± 15.8	0.002
SOFA score at admission†	5.9 ± 3.2	6.8 ± 3.3	5.8 ± 3.1	0.023
Mechanical ventilation	370 (85.8%)	64 (100%)	306 (83.3%)	< 0.001
Immunosuppression prior to admission to the ICU	42 (9.7%)	19 (29.7%)	23 (6.3%)	< 0.001
Corticosteroids for COVID Pneumonia	431 (100%)	64 (100%)	367 (100%)	1.0
Tocilizumab for COVID Pneumonia	82 (19%)	18 (28.1%)	64 (17.4%)	0.066
ICU-acquired bacterial superinfections	243 (56.3%)	60 (93.8%)	183 (50%)	< 0.001
CAPA probable	46(10.7%)	17(26.5%)	29(8%)	< 0.001
ICU LOS†	24.1 ± 27.3	49.7 ± 27.0	19.1 ± 24.5	< 0.001
ICU Mortality	78 (18.1%)	28 (43.8%)	50 (13.6%)	< 0.001
Hospital Mortality	81 (18.8%)	31 (48.4%)	50 (13.6%)	< 0.001

Data are shown as number (percentage). Abbreviations: SD: Standard Deviation; APACHE II: Acute Physiology and Chronic Health Evaluation II; SAPS II: Simplified Acute Physiology Score II; SOFA: Sequential Organ Failure Assessment; ICU: Intensive Care Unit; COVID: Coronavirus Disease; CMV: Cytomegalovirus; CAPA: COVID−19 Associated Pulmonary Aspergillosis; LOS: Length of Stay; ns: Not significant. † Expressed as media ± standard deviation

ICU-acquired bacterial superinfections were significantly more frequent in the CMV-COVID group compared to the non-CMV group (*p* < 0.001). We present the causal bacteria identified in CMV-COVID patients (Table 2).

**Table 2:** Causal Bacteria in ICU-acquired superinfections among CMV-COVID patients

Causal Bacteria	*n* = 60
*Enterococcus faecalis*	12 (20.0%)
*Enterococcus faecium*	11 (18.3%)
*Klebsiella pneumoniae*	10 (16.7%)
*Pseudomonas aeruginosa*	10 (16.7%)
*Stenotrophomonas maltophilia*	3 (5.0%)
*Burkholderia cepacia*	3 (5.0%)
*Escherichia coli*	3 (5.0%)
*Staphylococcus epidermidis*	3 (5.0%)
*Serratia marcescens*	2 (3.3%)
*Staphylococcus aureus*	2 (3.3%)
*Elizabethkingia*	1 (1.7%)

Data are shown as number (percentage). Abbreviations: ICU: Intensive Care Unit; CMV: Cytomegalovirus; COVID: Coronavirus Disease

CMV infection increased the risk of ICU mortality (OR 3.20; 2.19–4.67) and hospital mortality (OR 4.91; 2.76–8.75). Among the identified factors, CMV coinfection was the most significant independent risk factor for hospital mortality (Table 3).

**Fig. 2:**
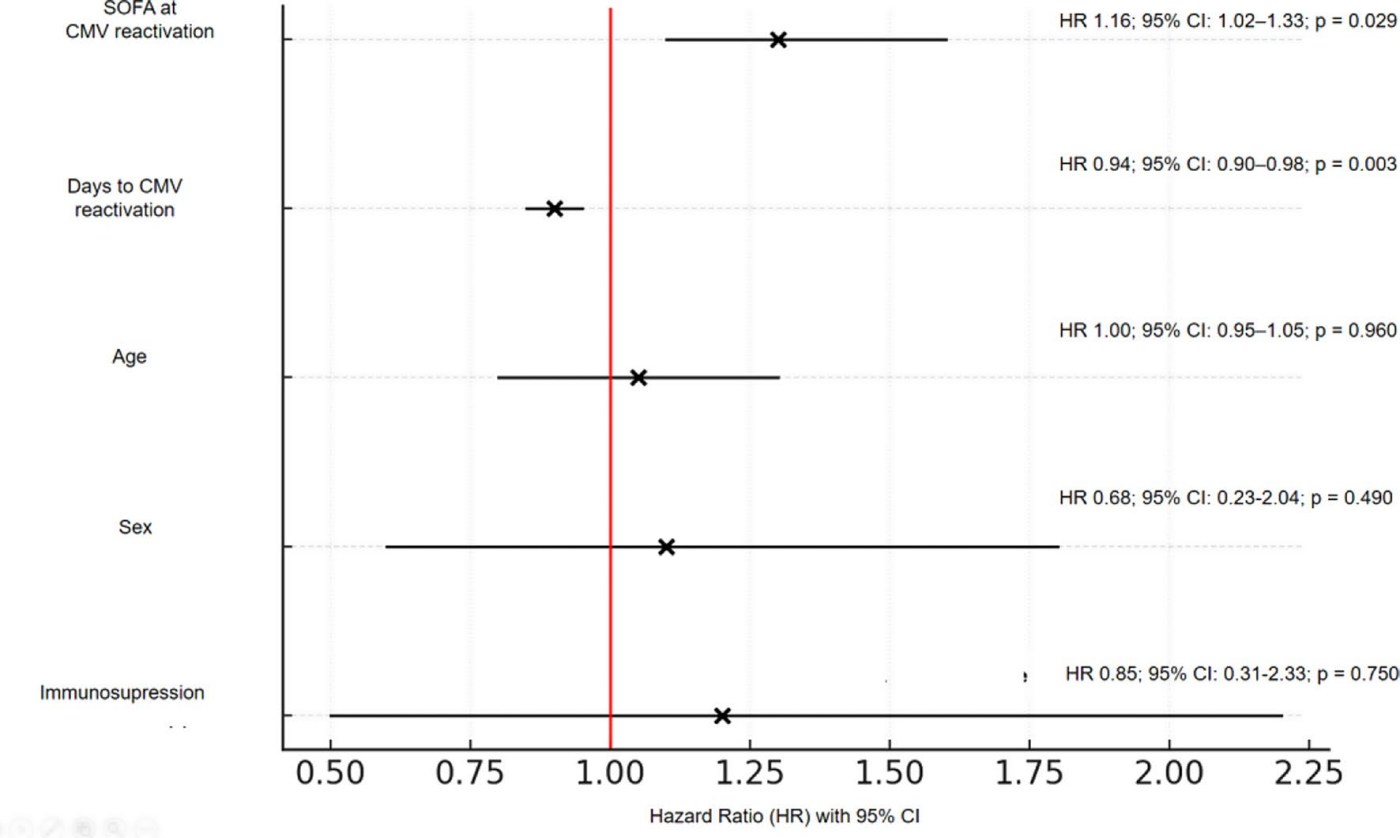
Forest plot of time-dependent Cox regression analysis for hospital mortality in CMV-COVID patients The reference line is at HR = 1; Abbreviations: HR: Hazard Ratio; CI: Confidence Interval; CMV: Cytomegalovirus; COVID: Coronavirus Disease; SOFA: Sequential Organ Failure Assessment

The time-dependent Cox regression analysis identified that a higher SOFA score at the time of CMV reactivation and earlier CMV reactivation were significantly associated with increased hospital mortality (Fig. 2).

### Characteristics and clinical entities in CMV-COVID

The median time to the first detection of CMV was 16 days (IQR 10.0–30.0). Figure 1 illustrates the distribution and mortality rates of the different CMV-COVID clinical entities: Asymptomatic viremia, CMV syndrome, and CMV disease, with mortality rates of 16%, 32%, and 57%, respectively (*p* = 0.087).

At the time of CMV-COVID diagnosis, 75% of patients had a viral load > 500 IU, and 53.1% had a load > 1000 IU. The majority (31/33) of patients with CMV disease presented pneumonia. CMV colitis was diagnosed by intestinal biopsy in 2 patients. The 16 patients with probable CMV pneumonia showed a viral load in BAL with a ratio of ≥ 2:1 BAL/plasma, with 25% (4/16) having a positive viral load in BAL but undetectable in plasma.

Specific treatment was given to patients with CMV syndrome or disease. Ganciclovir was the first-line antiviral in 98.2% (57/58) of patients.

**Table 3:** Multivariable logistic regression analysis with hospital mortality as the dependent variable

Variable	OR	95% CI	*p* value
Co infection CMV-COVID	4.91	2.76–8.75	0.019
Age > 60	4.11	2.31–7.33	0.065
SOFA at ICU admission > 5 points	2.31	1.27–4.19	0.004

Abbreviations: OR: Odds Ratio; CI: Confidence Interval; CMV: Cytomegalovirus; COVID: Coronavirus Disease; SOFA: Sequential Organ Failure Assessment; ICU: Intensive Care Unit

## Discussion

In our study, critically ill patients with SARS-CoV-2 pneumonia who developed CMV infection exhibited higher mortality rates compared to those without CMV infection.

The incidence of CMV infection under an active surveillance protocol in critically ill patients with COVID-19 was almost 15%. A recent study by Mattei et al., reported a CMV-COVID incidence of 61% in 120 COVID-19 ICU patients in Italy [16]. They attribute this high percentage to the high-risk profile of their cohort for CMV reactivation; however, the risk factors described are similar to those in our sample—critically ill COVID-19 patients treated with corticosteroids. Our findings align more closely with a recent meta-analysis of observational studies in critically ill patients, which reported a CMV-COVID incidence of 19% (95% CI, 13-28%) [17]. Routine CMV diagnosis is not standard practice in critically ill patients without hematologic malignancies. The implementation of an active surveillance protocol could help avoid underdiagnosis of this condition.

The association between SARS-CoV-2 infection and CMV infection could be explained by the complex immune system dysregulation seen in SARS-CoV-2, which includes both hyperinflammation and immunosuppression [18], combined with the immunosuppressive drugs used in treatment [19, 20].

We found significant differences in ICU and hospital mortality between the CMV-COVID group and the non-CMV group. An association between CMV infection and higher mortality was observed in our cohort of critically ill patients with SARS-CoV-2 pneumonia. Mattei et al. identified viral reactivation as a strong independent risk factor for hospital mortality (OR = 2.46, 95% CI: 1.02–5.89) [16]. In contrast, Gatto et al. conducted a multicenter study including 431 patients, 88 (20.4%) of whom had CMV reactivation. In this study, although hospital mortality was significantly higher in patients with CMV reactivation (67.0%) compared to those without (24.5%), the adjusted analysis did not confirm this association, suggesting that CMV-related mortality may rather reflect greater disease severity in these patients [21].

CMV-COVID group also showed significant differences in age, severity scores upon ICU admission, immunosuppression, need for respiratory organ support, higher rates of ICU-acquired co-infections, and length of stay. Hospital stay is an established risk factor for mortality, as shown in studies by Limaye [22] and Schöninger [3]. In our study, a multivariable analysis showed that CMV-SARS-CoV-2 co-infection remained an independent risk factor for hospital mortality (OR 4.91; 95% CI 2.76–8.75; *p* = 0.019).

Age > 60 years and SOFA score > 5 at ICU admission, were also independent risk factors for mortality. Schinas et al. (2023), in a systematic review, found that middle-aged and elderly patients with comorbidities are more likely to develop CMV co-infection [23]. Kalil et al. (2009) reported that the incidence of CMV infection is higher in patients with greater clinical severity, with rates of 32% in severe cases (APACHE II > 20, SAPS > 40, SOFA > 10) and 13% in less severe cases [2].

The time-dependent Cox regression analysis provides new insights into the potential association between CMV reactivation and mortality in critically ill COVID-19 patients. Our results suggest that the SOFA score at CMV reactivation may be independently associated with ICU mortality, underscoring the potential relevance of monitoring organ dysfunction progression during ICU stay.

Interestingly, earlier CMV reactivation was associated with increased hospital mortality, suggesting a potential link between early reactivation and higher mortality risk. However, to our knowledge, no studies have confirmed this association in critically ill CMV-COVID-19 patients, although similar findings have been documented in other populations, such as transplant recipients [24]. This emphasizes the importance of early CMV monitoring upon ICU admission, as timely detection and intervention could potentially mitigate its impact on patient outcomes. Further studies are needed to better understand the underlying mechanisms, although it is likely that immune dysfunction in critically ill patients, driven by CMV reactivation, could contribute to this increased risk. Specifically, CMV reactivation has been suggested to promote further immunosuppression through complex mechanisms involving TNF-alpha, interleukin-1 beta, and cellular immunity, potentially increasing susceptibility to secondary infections [25].

The median time to first detection of CMV was 16 days (IQR 10.0–30.0), similar to other studies [21]. There are no clear definitions for the clinical manifestations of CMV in critically ill SARS-CoV-2 patients, nor is there a consensus on treatment thresholds, complicating therapeutic decisions in a condition associated with high mortality [21, 23]. In this study, we adapted international definitions [7, 9] and observed a trend toward higher mortality with greater clinical severity, though without statistically significant differences, possibly due to the small sample size. The lack of clear definitions also affects the diagnosis of CMV pneumonia, which can lead to pulmonary fibrosis [26]. In 16 patients, CMV viral load in bronchoalveolar lavage (BAL) was at least double that in the blood, suggesting that the BAL-to-plasma ratio could be used as a diagnostic criterion.

Our CMV-COVID patients had a significantly higher number of ICU-acquired superinfections, consistent with other studies, such as Gatto et al. [21]. Enterococcal infections were the most frequently isolated pathogens, accounting for 38% of cases, a pattern that aligns with the high prevalence of *E. faecalis* and *E. faecium* reported in other critically ill COVID-19 patients [27]. Our findings support the observed association between CMV reactivation and increased susceptibility to secondary infections in this population.

The association between aspergillosis and COVID-19 has been well-documented. In our series, we found a significantly higher incidence of probable invasive pulmonary aspergillosis in CMV-COVID patients compared to the NON-CMV group (26.5% vs. 8%, *p* < 0.001). Caciagli et al. also reported a higher incidence of aspergillosis in patients with both COVID-19 and CMV [28]. These findings underscore the importance of actively screening for opportunistic microorganisms in COVID-19 patients [6].

Corticosteroids were not associated with an increased risk of CMV-COVID in our study, although this is difficult to analyze due to the high rates of corticosteroid use following the RECOVERY trial [12]. Similarly, tocilizumab was not associated with a higher risk of CMV infection.

We treated patients with CMV syndrome and disease using antivirals, with a mortality rate of 48.2%. In contrast, the mortality rate among untreated patients was 16%, although this only patient died from unrelated causes. The small number of cases limits the statistical significance of these results. There is no clear evidence of the efficacy of antiviral treatment in these patients, although treating those with specific CMV or HSV diseases and high viral load is appropriate [3, 21, 29]. These studies did not show clear mortality benefits from treating CMV reactivation in COVID-19 ICU patients, likely due to the heterogeneity of the treated population and the high prevalence of asymptomatic viremias. Based on these findings, our results support the approach of establishing a diagnosis according to proposed criteria and considering treatment primarily for cases of CMV syndrome and disease, as antiviral treatment for asymptomatic viremia has not been consistently associated with improved mortality outcomes.

### Limitations of the study

This single-center, observational study did not include a comparator group with a different antiviral strategy, limiting the generalizability of the results and the comparison of treatments. Additionally, the absence of a comparison group of critically ill non-COVID patients restricts our understanding of the specific impact of CMV-COVID co-infection.

Another important limitation is the potential for missed CMV diagnosis due to contraindications for bronchoscopy in some patients, which may have affected the detection of CMV in respiratory samples. Furthermore, the lack of consensus on the clinical definitions of CMV-related entities complicates diagnostic accuracy and could introduce variability in case classification.

A potential limitation of our study is the absence of baseline CMV serology data. However, considering the high CMV seroprevalence in the Spanish population [30], it is likely that most of our critically ill patients were CMV-seropositive upon ICU admission. While primary CMV infection cannot be entirely ruled out, it is rare in adults and may not have had a significant impact on our findings.

These limitations highlight the need for larger, multicenter, and comparative studies to validate our results, improve diagnostic criteria, and better understand the role of CMV reactivation in the progression of critical illness in COVID-19 patients.

## Conclusion

In our study, which implemented an active CMV surveillance protocol, a high incidence of CMV infection was observed among critically ill patients with SARS-CoV-2 pneumonia. CMV infection was associated with increased ICU and hospital mortality, and our analysis suggested it may be an independent risk factor for mortality in this population. An earlier reactivation of CMV appeared to correlate with a higher risk of hospital mortality. Furthermore, CMV co-infection was linked to a greater incidence of bacterial and fungal ICU-acquired infections, as well as prolonged hospital stays. The absence of standardized definitions and consensus regarding the clinical significance of CMV and treatment thresholds in COVID-19 patients poses challenges for therapeutic management. These findings highlight the need for further studies to provide additional data and help optimize the management of this condition.

## Data Availability

No datasets were generated or analysed during the current study.

## Abbreviations

CMV: Cytomegalovirus
ICU: Intensive care unit
SARS-CoV-2: Severe acute respiratory syndrome coronavirus 2
ARDS: Acute respiratory distress syndrome
BAL: Bronchoalveolar lavage
SOT: Solid organ transplant
OR: Odds ratio
HR: Hazard ratio
CI: Confidence interval
SOFA: Sequential organ failure assessment
SAPS II: Simplified acute physiology score II
APACHE II: Acute physiology and chronic health evaluation II
LOS: Length of stay
IU: International units
REDCap: Research electronic data capture
IPA: Invasive pulmonary aspergillosis
SPSS: Statistical package for the social sciences
SD: Standard deviation
